# Damages for Libel

**Published:** 1908-03-14

**Authors:** 


### Medico-Legal Points.

### DAMAGES FOR LIBEL.

### SUCCESSFUL ACTION BY A MEDICAL MAN.

The case which the other day came before Mr. Justice Darling in the Court of King's Bench, when Dr. Morris James Williams, of Walham Green, was awarded £500 damages and costs against the *Star* newspaper could, in our opinion, have had no other logical result.

Towards the end of 1906, Dr. Williams, on being called in to attend Mrs. Blume, of Maxwell Road, Fulham, found the latter already dead, after having made a will in favour of Richard Brinkley, who was subsequently convicted and executed in connection with the Croydon poisoning case. After a post-mortem examination of Mrs. Blume's body Dr. Williams came to the conclusion that death was due to apoplexy, and the inquest resulted in a verdict of death from natural causes. Some months later, however, when Brinkley in the interval had been arrested, Mrs. Blume's body was exhumed and another examination was made by Sir Thomas Stevenson and Dr. French in Dr. Williams's presence.

The *Star* reported that this examination was abortive because some parts of the organs which it was desired to examine were missing. Sir Thomas Stevenson, however, at the trial said he had found all the organs necessary to discover whether poison did or did not exist in the body, and no trace of it was found. One is reminded by this case of a page in Taylor's *Medical Jurisprudence*, where that great authority points out the danger of allowing a suspected individual to be present at the post-mortem on the remains of a supposed victim. The case of the famous Rugeley poisoner, Dr. Palmer, is cited as one in point. Although ugly whispers were then current, yet Palmer was not only present at the examination of the body of Cook, for whose murder he was afterwards convicted and hanged, but during the post-mortem he actually with his penknife slit the skin covering one of the jars sealed up for analysis. Another instance is given as having occurred in France, where an individual was strongly suspected of having murdered another, whose life was heavily insured in his favour, by administering a peculiar poison, taking effect on a particular organ. So greatly were the French authorities exercised over this mystery that they sought, and obtained, Dr. Taylor's assistance at the analysis of the organs of the dead man. Unfortunately, however, the suspect had been permitted to be present at the post-mortem, after which the particular organ in question was found to be missing, and the result was that the authorities could take no action, and the insurance companies had to pay heavy sums to the suspected heir.

There is, of course, no parallel between the two cases just mentioned and that of the second post-mortem on Mrs. Blume, and, therefore, no shadow of justification for the *Star* libel. True, Dr. Williams was a professionally interested party, though not in the same sense as in the two remarkable cases we have quoted, which may possibly have been in view when the libel so properly punished was written.

